# Targeting of Evolutionarily Acquired Cancer Cell Phenotype by Exploiting pHi-Metabolic Vulnerabilities

**DOI:** 10.3390/cancers13010064

**Published:** 2020-12-28

**Authors:** Bryce Ordway, Michal Tomaszewski, Samantha Byrne, Dominique Abrahams, Pawel Swietach, Robert J. Gillies, Mehdi Damaghi

**Affiliations:** 1 Department of Cancer Physiology, Moffitt Cancer Center and Research Institute, Tampa, FL 33612, USA; bryce.ordway@moffitt.org (B.O.); michal.tomaszewski@moffitt.org (M.T.); samantha.byrne@moffitt.org (S.B.); dominique.abrahams@moffitt.org (D.A.); robert.gillies@moffitt.org (R.J.G.); 2 Department of Physiology, Anatomy & Genetics, University of Oxford, Oxford OX1 3PT, UK; pawel.swietach@dpag.ox.ac.uk; 3 Department of Oncologic Sciences, Morsani College of Medicine, University of South Florida, Tampa, FL 33612, USA

**Keywords:** evolutionary therapy, darwinian evolution, tumor microenvironment, cancer cells subpopulations, diclofenac, koningic acid, spheroid, 3D co-culture

## Abstract

Evolutionary dynamics can be used to control cancers when a cure is not clinically considered to be achievable. Understanding Darwinian intratumoral interactions of microenvironmental selection forces can be used to steer tumor progression towards a less invasive trajectory. Here, we approach intratumoral heterogeneity and evolution as a dynamic interaction among subpopulations through the application of small, but selective biological forces such as intracellular pH (pHi) and/or extracellular pH (pHe) vulnerabilities. Increased glycolysis is a prominent phenotype of cancer cells under hypoxia or normoxia (Warburg effect). Glycolysis leads to an important aspect of cancer metabolism: reduced pHe and higher pHi. We recently showed that decreasing pHi and targeting pHi sensitive enzymes can reverse the Warburg effect (WE) phenotype and inhibit tumor progression. Herein, we used diclofenac (DIC) repurposed to control MCT activity, and Koningic acid (KA) that is a GAPDH partial inhibitor, and observed that we can control the subpopulation of cancer cells with WE phenotype within a tumor in favor of a less aggressive phenotype without a WE to control progression and metastasis. In a 3D spheroid co-cultures, we showed that our strategy can control the growth of more aggressive MDA-MB-231 cells, while sparing the less aggressive MCF7 cells. In an animal model, we show that our approach can reduce tumor growth and metastasis. We thus propose that evolutionary dynamics can be used to control tumor cells’ clonal or sub-clonal populations in favor of slower growth and less damage to patients. We propose that this can result in cancer control for tumors where cure is not an option.

## 1. Introduction

The initiation and development of cancers is associated with major metabolic alterations in response to dynamically changing microenvironmental conditions such as hypoxia and acidosis [1]. These forces will select for the fittest phenotype in the context of the current microenvironment. Cancer cells that are more plastic and adaptable can acclimate to an increasing array of emergent microenvironmental selective forces. Combined, these factors define tumors as highly dynamic ecosystems in which many different cancer cell subpopulations compete for space and resources [2]. The microenvironment around the cells can alter the local fitness of cancer cell subpopulations in a tumor; leading to possible dramatically different evolutionary trajectories selecting for cells with different phenotypic and genotypic properties [3]. We have previously reported how acid-producing and acid-resistant phenotypes can engineer the tumor ecosystem in order to increase their own fitness, allowing them to take over the population [3,4]. We have also showed the different strategies that cancer cells acquire to adapt to their microenvironment [3]. Here, we investigate the impact of switching off certain phenotypes that give cancer cells a competitive advantage. By switching these off, we can influence the distribution of sub-populations with the end game of turning the majority of the population into a less aggressive phenotype with decreased invasion and metastasis. The idea is to take away any evolutionarily acquired or selection driven advantages of the more aggressive clones in population to allow for non-aggressive cells to be able to compete [5].

Glycolysis is the prominent phenotype of cancer cells under hypoxia or normoxia (Warburg effect). Glycolysis produces acid, which must be removed from cells, resulting in an important aspect of cancer metabolism: i.e., reduced extracellular pH (pHe~6.5–6.7) and increased intracellular pH (pHi ≥ 7.2). For cells to compete they must adapt to these conditions and thus these adaptations can be used against them as a vulnerability [6]. Adaptive mechanisms include: increased expression and activity of acid extruders, such as monocarboxylate transporters (MCT) including: MCT1 and MCT4, Na^+^-H^+^ exchanger 1 (NHE1) and V-ATPases, as well as membrane bound exofacial carbonic anhydrases such as CA9 or CA12 to maintain an optimal pHi [6]. An optimal pHi gives cancer cells some proliferative advantages [6]. Herewith, we use two drugs that we are repurposing to target pHe and pHi metabolic adaptations so that the more aggressive cancer cell sub-populations lose their selective advantage over the less aggressive phenotypes. The first drug is diclofenac, a clinically used non-steroidal anti-inflammatory drug (NSAID), that has been shown to have an inhibitory effect on MCTs [7,8]. Targeting MCTs is a promising approach that many groups are following to find treatment against the glycolytic phenotype of cancer cells such as MCT1/2 inhibitors [9]. However, these drugs have not yet been successful in treating cancer, as cells have shown the ability to switch isoforms from e.g., MCT1 to MCT4 [6,9]. Diclofenac is putatively a pan-MCT inhibitor [7]. 

The second drug is koningic acid (KA), a natural product produced by fungi, which inhibits the function of GAPDH [10,11].

In our previous work of systems analysis of cancer cells’ metabolic vulnerabilities [6], we observed that GAPDH is highly pHi sensitive and has a key role in promoting a WE phenotype. This pH sensitivity of GAPDH means that the effects of its inhibition on cell proliferation are enhanced at an acidic intracellular pH. This acidic pHi vulnerability is the reasoning for coupling with diclofenac, which we demonstrated to be a potent inhibitor of MCT activity, causing decreased intracellular pH. Other groups also reported GAPDH as a selective WE phenotype target [10] that can be partially and irreversibly inhibited by KA. 

Evolutionary-based therapies use Darwinian principles to delay the proliferation of aggressive populations regardless of their genotype states [3,5,12]. These therapies can be guided by the use of mathematical models of cell population dynamics based on competing mechanisms of cancer cell sub-population within the tumor [13,14]. Conventional maximum dose therapy (MDT) permits an unopposed proliferation of resistant and/or more plastic cells by removing sensitive and/or less plastic cancer cell populations, a phenomenon known as “competitive release” in ecology [5]. With “competitive release”, clonal and sub-clonal population balance is not maintained amongst all different clones. Alternatively, evolution-based therapies use Darwinian principles to delay the proliferation of resistant populations [3,5], maintaining clonal and sub-clonal population balance. A form of evolutionary therapy is adaptive therapy (AT), where the competitive release of resistant clones is delayed by the application of intermittent or dose-varying therapeutic regimens, to maintain a balance between sensitive and resistant clones to successfully control tumor growth [5,15,16]. In our approach, we exploit metabolic adaptations to acidosis as a vulnerability for the more aggressive tumor cells in order to cancer growth. We selected our representative cell lines, less aggressive (MCF7) and more aggressive (MDA-MB-231), based on the previously described phenotypes [17,18,19,20,21]. 

## 2. Results

### 2.1. Diclofenac and Koningic Acid Reduce the Glycolytic Activity of Cancer Cells

**Figure 1:**
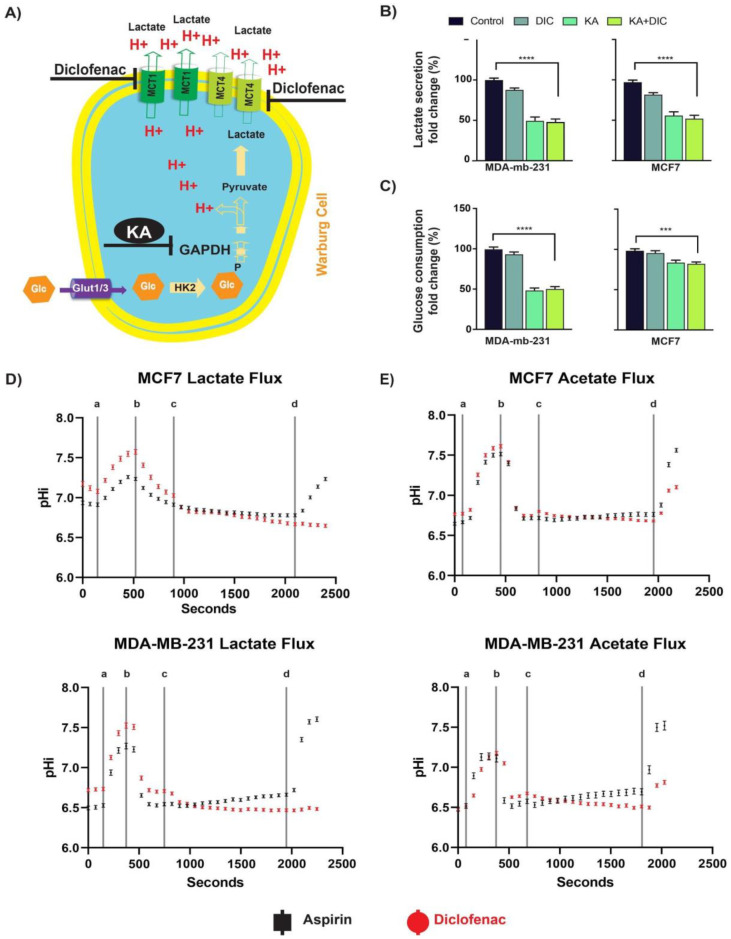
Diclofenac and koningic acid reduce the glycolytic activity of cancer cells. (**A**) Schematic showing effect of Diclofenac (1 μM) to reduces the activity of MCT transporters leading to acidification of the intracellular pHi. Koningic acid (KA) reduces glycolytic activity by inhibiting GAPDH that has an alkaline pH optimum. (**B**) Lactate production was reduced in both MCF7 and MDA-MB-231 cells after treatment with diclofenac, KA, or both. (**C**) Glucose consumption was reduced in both MCF7 and MDA-MB-231 cells after treatment with diclofenac, KA, or both. (**D**) MCF7 and MDA-MB-231 cells were treated with diclofenac or Aspirin to measure the effect of drug on their lactate transport abilities. Line (a) represents the switch from a 30 mM lactate environment to a 0 mM Lactate environment. Line (b) represents the switch from a 0 mM lactate environment to a 30 mM lactate environment. Line (c) represents the switch from a 30 mM lactate environment − drug treatment to a 30 mM lactate environment + drug treatment. Line (d) represents the switch from a 30 mM lactate environment + drug treatment to a 0 mM lactate environment + drug treatment. (**E**) MCF7 and MDA-MB-231 cells were treated with diclofenac or Aspirin to measure the effect of drug on their pHi altering effects. Line (a) represents the switch from a 30 mM acetate environment to a 0 mM acetate environment. Line (b) represents the switch from a 0 mM acetate environment to a 30 mM acetate environment. Line (c) represents the switch from a 30 mM acetate environment − drug treatment to a 30 mM acetate environment + drug treatment. Line (d) represents the switch from a 30 mM acetate environment + drug treatment to a 0 mM acetate environment + drug treatment. *p*-values are represented as follows: *** *p* < 0.001, **** *p* < 0.0001.

H^+^-monocarboxylate transporters (MCTs) play a major role in transporting organic anions such as lactate across cell membranes. Since the transport cycle is coupled to H^+^ ions, the activity of MCTs also affects intracellular pH (pHi) within cells [22]. It has been shown that MCT inhibition decreases cancer cells’ aggressive phenotypes [9,23,24] by reducing the lactate efflux, leading to acidification of pHi and buildup of intracellular [lactate] [6]. It has been shown that diclofenac can inhibit MCTs [7] and that koningic acid (KA) can inhibit GAPDH [10,25,26], which is very sensitive to pHi [6] (Figure 1A). As proof of principle, we measured the effect of diclofenac and KA on glucose consumption and lactate production of MCF7 and MDA-MB-231 breast cancer cell lines. In both cell lines, diclofenac and KA reduced the lactate production (Figure 1B) and glucose consumption (Figure 1C) significantly within 72 h of treatment. Notably the effect was larger in the more highly glycolytic MDA-mb-231 cells. We then measured the activity of both drugs in the 3D culture of MCF7 and MDA-MB-231 spheroids over time. We observed that both drugs showed effects as early as 24 h (Figure S1A,B). These results indicated that both drugs are effective in inhibiting glycolysis as proposed and can work both in 2D and 3D experiments. We also measured the glutamine and glutamate concentration in the media following treatments of both cell lines with both drugs under normoxia and hypoxia and found no differences among the groups (Figure S1C). 

### 2.2. Diclofenac Inhibits MCT Activity

To test whether the effect of diclofenac treatment on glycolysis was due to inhibition of lactate export, we measured MCT activity before and after treatment with diclofenac using pHi as a reporter of transmembrane flux. To rule out if the effect on pHi change is because of NSAID activity of diclofenac, we also measured the MCT activity on the same cells treated with aspirin. Using a superfusion microscopy system and cells loaded with the pH reporter dye SNARF-1, we measured the time course of pHi during superfusate maneuvers that trigger net transmembrane flux of lactate (Figure 1D). When presented with extracellular lactate, cells acidified, and when extracellular lactate was withdrawn, cells alkalinized. The rate of these pHi changes are measures of MCT activity. In the presence of diclofenac, however, efflux of lactate was completely inhibited in both cell lines. Controls included another NSAID, aspirin, which was without effect. As these are acute effects, cell death was not expected to play a role (vide infra), and we further ruled it out, due to the inability of dead cells to retain the SNARF-1 dye. To confirm that diclofenac was acting on a specific transport process, i.e., MCT, rather than causing a generalized tightening of membrane permeability, experiments were repeated with acetate in place of lactate. Compared to lactate, acetate can permeate the cell membrane in an MCT-independent manner by partitioning across the lipid matrix as acetic acid, and its efflux was unaffected by diclofenac (Figure 1E). This suggests that the action of diclofenac is targeted to MCT, the main route for lactate traffic in and out of cells. The results of this assay are not indicative of what is expected to be seen under physiological conditions. The large changes in pH observed in these experiments are not sustained for long periods of time, and are induced by superphysiological lactate levels. The purpose of these experiments was solely to determine the effects of Diclofenac on a cells ability to export lactate; not to demonstrate specific pHi values under drug treatment. 

### 2.3. Diclofenac and Koningic Acid Decrease the Viability of Cancer Cells

**Figure 2:**
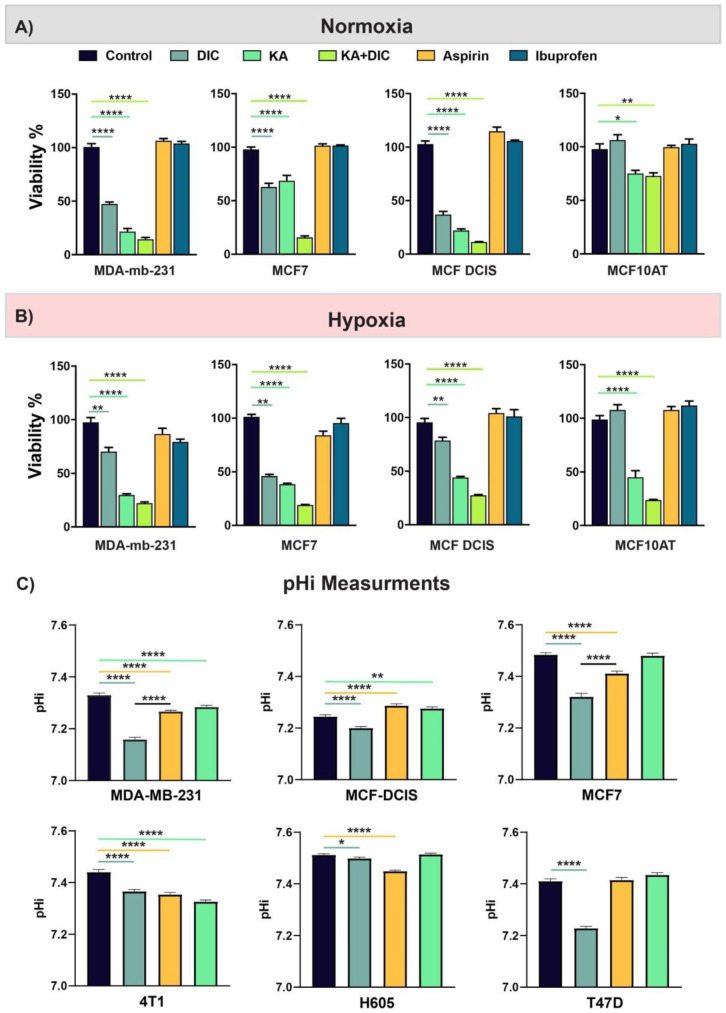
Diclofenac and koningic acid decrease the viability of cancer cells. (**A**,**B**) Viability of different breast cancer cell lines (MDA-MB-231, MCF7, MCF DCIS, MCF10AT) were measured in 2D using CCK8 kit under normoxia (**A**) and hypoxia (**B**) and different treatments. Diclofenac combination with GAPDH inhibition had the highest effect on cell viability. Interestingly other NSAID drugs such Aspirin or Ibuprofen didn’t have any effect on cell viabilities. Error bars plotted as SD. (**C**) Intracellular pH measurement of MDA-MB-231, MCF-DCIS, MCF7, 4T1, H605, and T47D cancer cells using SNARF1, after 16 h treatment. Diclofenac decreased the pHi significantly in all cell lines. Error bars plotted as SEM. *p*-values are represented as follows: * *p* < 0.05, ** *p* < 0.01, **** *p* < 0.0001.

To test if the effects on glycolysis translate into the effective killing of cancer cells, we assessed the survival of cells using CCK8 viability assays and CelltiterGlo (see Methods) in both hypoxia and normoxia. We experimented with both normoxic and hypoxic conditions as they both commonly occur in the microenvironment of solid tumors such as breast cancer, and cell viability will be expected to be much more sensitivity to glycolysis inhibition under hypoxic conditions. In solid tumors, cells that are further than 160 micrometers from the vasculature they experience a hypoxic condition that makes the cells glycolysis dependent [27,28]. Many cancer cells overexpress or over-activate MCTs to deplete the lactate produced under the hypoxic conditions, although this response is not universal as shown in supplemental Figure S2. Under normoxia (Figure 2A) and hypoxia (Figure 2B) cell survival was measured after treatment with diclofenac, KA, or both in MCF7 and MDA-MB-231 cells. As shown in Figure 2A, diclofenac alone reduced the viability of MDA-mb-231, and MCF10/DCIS cells, which are highly glycolytic and tumorigenic, as well as MCF-7 cells which are tumorigenic and only moderately glycolytic. Notably there was no effect on viability of MCF10AT cells, which are neither tumorigenic nor glycolytic. More pronounced effects with a similar distribution were observed with KA and the combination of DIC + KA. Surprisingly, the effect of DIC on viability under hypoxia (Figure 2B) was less significant cf. normoxia (Figure 2A), whereas the effects of KA and the DIC + KA combination were no different. To test the activity of the drug on viability and H^+^ export flux in the same experiment we performed a viability assay while monitoring the acidification of conditioned media. Inhibition of MCTs or GAPDH should reduce rate of media acidification. After treatment and before viability assays we measured the pHe of the media using the Five Easy^TM^/FiveGo^TM^ pH meter (Mettler Toledo, Columbus, OH, USA see Methods). The acidification was reduced by both drugs and the pH of media was neutral, validating the effect of both diclofenac and KA on lowering glycolytic activity of cells (Figure S3). 

To determine the effect of diclofenac and KA on the pHi of cancer cells, we measured the pHi of a panel of breast cancer cell lines (MDA-MB-231, MCF-DCIS, MCF7, 4T1, H605, and T47D), loaded with SNARF-1-AME (Figure 2C). For 16 h, cells were treated with diclofenac (100 µM), KA (1 µM), or aspirin (300 µM), and then imaged in buffered RPMI media. All cell lines exhibited a significantly lower pHi with the treatment of diclofenac, and 4 of the 6 diclofenac treated cell lines had significantly lower pH than the aspirin group. 

Considering the different microenvironment in solid tumors such as hypoxia and acidosis and their combination we wanted to test if inhibiting MCTs and GAPDH have an effect on the viability of cancer cells. These conditions are also very important to be targeted because they are specific for tumors and not experienced by normal cells. Therefore, we performed the survival assays on both MCF7 and MDA-MB-231 cells under four possible combinations of change in oxygen level and pH as following: (i) Normal pH-Normoxia, (ii) Low pH-Normoxia, (iii) Normal pH-Hypoxia, and (iv) Low pH-Hypoxia. Targeting MCTs and GAPDH under different microenvironments reduces the cancer cell’s viability (Figure S4). The effect was greatest with the combination of both drugs and under the most unique condition of solid tumors -hypoxia, and acidic pH (Figure S4). 

### 2.4. Diclofenac and Koningic Acid Reduce the Warburg Phenotype of Cancer Cells

**Figure 3:**
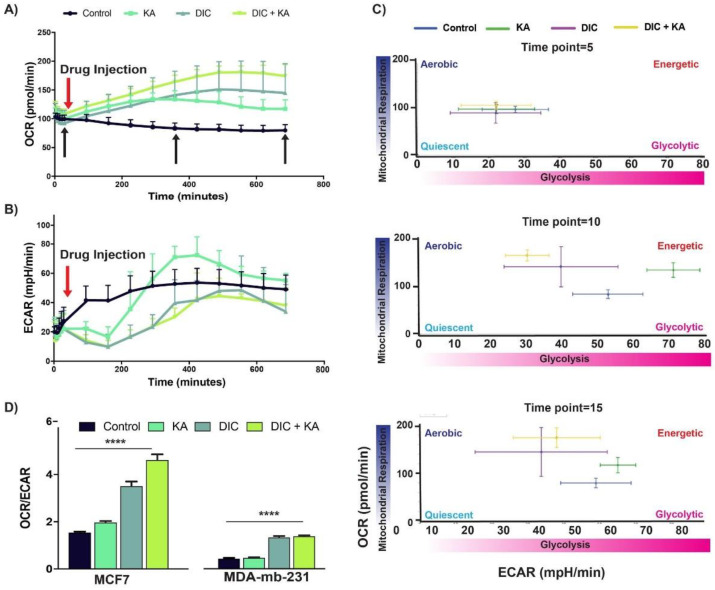
Diclofenac and koningic acid reduce the Warburg phenotype of cancer cells. (**A**) Oxygen Consumption Rate (OCR) and (**B**) Extracellular acidification rate (ECAR) of MCF7 cancer cells treated with, KA, diclofenac, and combination of both in real-time. (**C**) Energy map extracted from A and B under different treatments. A combination of KA and diclofenac had the highest effect on the reduction of WE phenotype in MCF7 breast cancer cells. (**D**) Glycolytic rate assay of both MCF7 and MDA-MB-231 cells confirmed the maximum reduction of WE phenotype with the combination of KA and diclofenac for 72 h. All the seahorse experiments were done in 6 replicates per condition and the data is normalized to the protein concentration of the cells per each well. The data is represented as the mean with the error bars as SD. *p*-values are represented as follows: **** *p* < 0.0001.

The Warburg effect (WE) phenotype is defined by a high glycolytic rate even in the presence of oxygen (aerobic glycolysis) and is associated with the progression and aggressiveness of cancers [29,30,31,32]. We recently showed that inhibiting proton pumps such as MCTs and pH-sensitive glycolytic enzymes such as GAPDH or GPI, reduces the WE phenotype of cancer cells [6]. Here we used the same strategy to reduce the WE phenotype by repurposing diclofenac from a NSAID to an MCT inhibitor and a natural product produced by fungi, koningic acid to target GAPDH. Above, we already showed that these compounds can reduce the glycolytic phenotype of cancer cells, and here we are using Seahorse measurements to confirm the inhibitor effect of these products on aerobic glycoysis. Seahorse can measure the glycolytic condition through simultaneous of proton efflux (ECAR) and mitochondrial respiration (OCR), which can be used to assess WE phenotype [6,33]. In the first experiment we interrogated at the real-time effect of diclofenac of KA on ECAR and OCR of MCF7 cancer cells (Figure 3A,B). KA and diclofenac monotonically increased the OCR compared to control and the combination of the two had the greatest effect (Figure 3A). KA and diclofenac both caused an initial drop of ECAR that lasted for 150 min that slowly returned to control levels. The recovery of ECAR to KA was more rapid compared to either conditions that contained diclofinac, which may be due to differences between GAPDH and MCT regulation (Figure 3B). This could imply that even if the cells over-activate or overexpress the GAPDH, because the pHi is too low the phenotype can’t be survived. We also measured the WE phenotype (ECAR/OCR) of these cells in three different time points: (i) right before drug injection, (ii) 5 h after injections, and (iii) 12 h after injections (black arrows in Figure 3A). These results showed that a combination of KA and diclofenac has the highest effect on decreasing the Warburg phenotype of cancer cells in mentioned time points (Figure 3C). To confirm this finding, we also performed a Seahorse experiment based on glycolytic rate assay that measured the maximum glycolytic capacity of cells by, first, shutting down mitochondrial respiration and then glycolysis (Methods). This assay also showed the highest inhibition of WE phenotype by the combination of KA and diclofenac in both MCF7 and MDA-MB-231 breast cancer cells (Figure 3D). 

### 2.5. Reducing Warburg Phenotype Can Change Population Dynamics

Cancer evolution is a result of genetic diversification and clonal selection within the adaptive landscapes of tissue ecosystems. Therapeutic approaches can destroy some cancer clones, but it may also create a new space and different selective pressure resulting in expansion of resistant and most probably more aggressive clones aka “competitive release” in ecology and evolution. Evolutionary based therapeutic design can be used to control cancers when a cure is not possible. For that reason, understanding Darwinian intratumoral dynamics and their interactions with microenvironmental selection forces is critical to steer a tumor into a less invasive phenotype.

**Figure 4:**
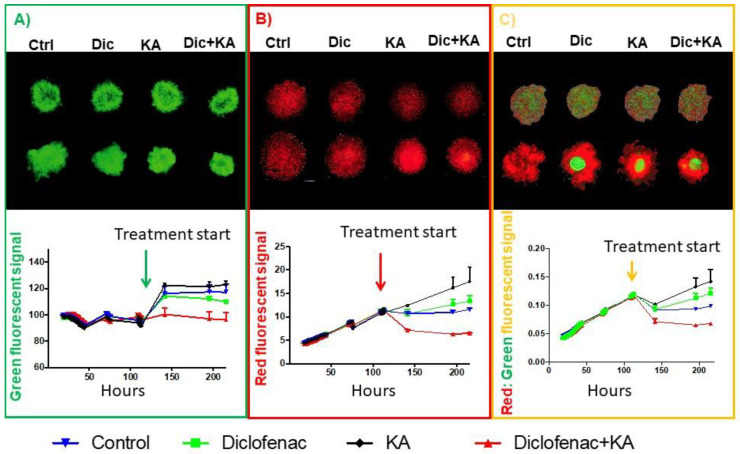
Spheroid 3D co-culture shows the role of evolutionary designed treatment in tumor cells population dynamics. (**A**) Images of the spheroids of MCF7 cells that were fluorescently tagged with GFP in monoculture. (**B**) Images of spheroids of MDA-MB-231 cells fluorescently tagged with RFP in monoculture. (**C**) Co-culture of MCF7-GFP and MDA-MB-231-RFP. The ratio in co-culture is 1:1 for experiment initiation. The top row are the pretreatment images and the bottom raw for each condition is the last time point images. Underneath the images is a fluorescent signal analysis of the spheroid experiment under all four treatment conditions. Each experiment has 8 replicates. The data are represented as mean with the error bars as SD.

In 3D spheroid experiments we investigated the competitive release strategy to develop a new treatment design. Two distinct cell types were used to provide insight into the dynamic interactions among tumor cell subpopulations, a crucial element of intratumor heterogeneity. Mixed-culture competition experiments in spheroids can successfully track competitive outcomes amongst different cell types [34]. In 3D spheroid experiments differences in growth rates, carrying capacities and competition coefficients can be determined accurately [34]. For this experiment, we mono- and co-cultured MCF7-GFP and MDA-MB-231-RFP cells (1:1 ratio) and treated them with DMSO (control), diclofenac, KA, or combination of KA and diclofenac (Figure 4A–C). As a control experiment we grew spheroids in different microenvironmental conditions and with different ratios of MCF7 to MDA-MB-231 cells. In any microenvironmental condition and with any ratio of the two cancer cells, MDA-MB-231 cells always eventually dominated the MCF7 cells and took over the whole population (Figure S5). After treating the spheroids with diclofenac, KA, or the combination, we showed that these metabolically designed drugs can steer the population dynamics towards the less aggressive phenotype cells (MCF7-GFP). Figure 4A,B are controls showing effect of the agents on monocultures. Figure 4C shows that, in co-culture conditions, the green signal that belongs to MCF7-GFP was increased in response to both drug and the combination, compared to the control group. These results show that it may be possible to control population dynamics in tumors based on their metabolic vulnerability.

### 2.6. Evolutionary Designed Therapy Controls Tumor Growth and Metastasis In Vivo

**Figure 5:**
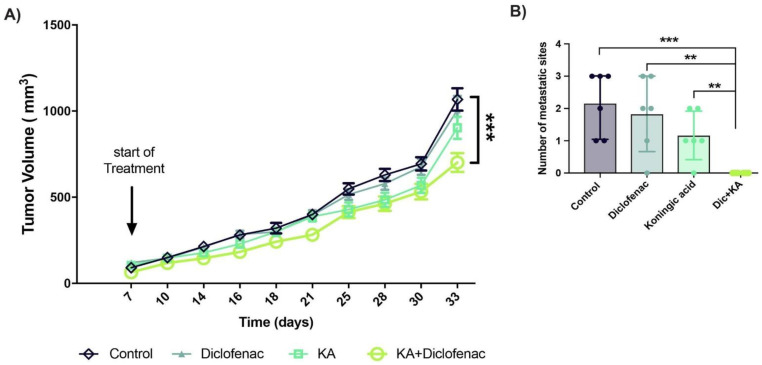
Animal experiments confirm the efficiency of evolutionary designed therapy. (**A**) Female NSG mice injected with a mixture of 5 million MCF7 and 5 million MDA-MB-231 breast cancer cells and randomized into four groups of 6 and treated with, DMSO, diclofenac, KA, and combination. Tumor size is measured every two or three days by calipers and also once a week with ultrasound. Data is presented is measurements by calipers and are mean with SD as their error bars. (**B**) The number of metastasis sites in vital organs such as liver, lungs, kidney, and spleen at the endpoint. The data is represented as mean with the error bars as the SD. *p*-values are represented as follows: ** *p* < 0.01, *** *p* < 0.001.

To translate our in vitro 2D and 3D results to in vivo we then performed animal experiments in female NSG mice. The experimental design was similar to the 3D spheroid experiment co-culture -MCF7 and MDA-MB-231 cells were mixed in 1:1 ratio and inoculated into the mice mammary fat poad. Mice were randomized into four groups of six mice per group and treated with diclofenac, KA, both, or none (DMSO). Primary tumor growth was unaffected by either monotherapy cf. controls, but there was a significant decrease caused by the combination of diclofenac and KA (Figure 5A). 

At the end of the experiment we extracted the tumors and all the vital organs from each mouse and scored for metastasis. Notably, the diclofenac + KA combination group was metastasis-free, while all other groups had measurable metastases across multiple sites (Figure 5B). These results indicate that evolutionary therapeutic approaches based on metabolic targeting can be used to control tumor growth and even change aggressive phenotypes such as metastasis.

## 3. Discussion

Tumor evolution follows the Darwinian principles; meaning nature selects for phenotype not genotype [5]. When cancer initiates and develops, many clones and sub-clones emerge that are genetically heterogeneous with many of them presenting the same phenotype such as the Warburg effect (WE) phenotype [35]. The emergence of one phenotype from many clones with different genetic backgrounds implies the fitness of the selected phenotype to the selection forces. The WE phenotype is associated with progression and invasion of many cancers and is defined by a high glycolytic rate in the absence or presence of oxygen (aerobic glycolysis). Most cancer cells reprogram their metabolism in favor of aerobic glycolysis despite the presence of plentiful oxygen in their microenvironment, implying higher fitness of WE phenotype. 

During the evolution of tumor cells and through adaptation to constantly variable microenvironment tumor cells adapt and acquire phenotypes helping them survive and grow. These acquired characteristics can be used as a new vulnerability against them. As mentioned previously, we recently showed that certain targets for inhibition of glycolysis have increased effects of inhibition at a lower intracellular pH [6]. To test this principle, we coupled Diclofenac, a compound we demonstrated to be a potent inhibitor of MCT’s, with Koningic Acid, a known inhibitor of GAPDH. The MCT inhibition by Diclofenac provided the decrease in phi needed to enhance the effectiveness of KA. In this paper we showed that targeting the WE phenotype in more aggressive cancer cells can take their competitiveness from them so the less malignant cells can compete with them in a tumor niche. We used a simple model of co-culture of two cell lines with extremely opposing characteristics: MDA-MB-231 cells that are triple-negative fast proliferating, tumorigenic and metastatic in mouse xenograft models that are also highly glycolytic with WE phenotype and MCF7 cells that are ER/PR positive, slow-growing and tumorigenic in mouse xenografts with the help of estrogen pellets but not metastatic and very low glycolytic and not WE phenotype. The co-culture of these two cell lines was used as a simple population model of tumor heterogeneity to monitor the population dynamics of the tumor. In the future, these findings will be confirmed and expanded in a more complex, transgenic tumor model, to observe the effect of real intratumor heterogeneity in cell subpopulation. Using our metabolic targeted strategy, we showed that it is possible to control the population dynamic and heterogeneity. This population dynamic adjustment was able to successfully control tumor growth and metastasis in the mouse xenograft model. Therefore, we propose a novel strategy of cancer control for the tumor where a cure is not an option. We propose evolutionary principles of tumor growth that can be used to control tumor cells’ clonal or sub-clonal population in favor of slower growth and less damage to patients.

## 4. Methods

### 4.1. Cell Culture

MCF-7 and MDA-MB-231 cells were acquired from American Type Culture Collection (ATCC, Manassas, VA, USA, 2007–2010) and were maintained in DMEM-F12 (Life Technologies, Carlsbad, CA, USA) supplemented with 10% fetal bovine serum (HyClone Laboratories, Logan, UT, USA). Cells were tested for mycoplasma contamination and authenticated using short tandem repeat DNA typing according to ATCC’s guidelines. Cells were treated with diclofenac (1 μM) and/or koningic acid (1 μM) dissolved in the media of the cells.

### 4.2. Hypoxia Cell Culture

For hypoxia conditions we used a Biospherix X-Vivo Hypoxia Chamber. The conditions in each hypoxia chamber kept at 37 °C, 5% CO_2_, with 0.1% O_2_ and 94.9% N_2_ or 1.0% O_2_ and 95% N_2_. The chamber was quality controlled and calibrated according to the manufacturer’s specifications (Biospherix, Parish, NY, USA). 

### 4.3. Transfection (GFP/RFP Plasmids)

To establish stable cell lines, the MCF-7 and MDA-MB-231 cells were infected with Plasmids expressing RFP or GFP using Fugene 6 (Promega, Madison, WI, USA) at early passage and were selected using 2 µg/mL puromycin (Sigma, St. Louis, MO, USA). 

### 4.4. Mettler Toledo Five Easy^TM^/FiveGo^TM^ pH Meter

The pH meter was first calibrated by using standard pre-made calibration buffer solutions for pH 4, 7, and 10. Then using the pH probe the pH of the control media used to grow the spheroids was measured. This pH was used as a baseline to compare the pH of the pHe media of the spheroids. 

### 4.5. Viability Assays

#### 4.5.1. CCK8

Cell viability was measured after treatment with drugs (diclofenac and koningic acid or both) and DMSO as control using Cell Counting Kit-8 (CCK-8) under different microenvironmental conditions such as normoxia, hypoxia, normal pH, or low pH. CCK8 is a sensitive colorimetric-based viability assay based on Dojindo’s highly water-soluble tetrazolium salt, with WST-8 as its active agent. CCK8 was used to measure viability as it is not pH sensitive and can be added to the cells directly in their niche, without fixation or change of media. For measuring viability, cells were seeded in a 96-well plate (with triplicate of the same samples), and viability was measured at the indicated intervals. The experiments were repeated at least two times.

#### 4.5.2. CellTiter-Glo^®^

This 3D Cell Viability Assay is designed to determine the number of viable cells in 3D cell culture based on quantitation of the ATP through based on Luminescent Cell Viability Assay chemistry. The lytic capability of the reagents is much higher than the 2D assay and the assay is compatible with 96 well-plate formats of our spheroid experiments that make it ideal for high-throughput screening. In our assay 72 h after treatment of spheroid with inhibitors, the lysis buffer directly added to each well containing one sphere of MDA-MB-231 or MCF7 cells. The plates were incubated in RT for 10 min on a rocking shaker. Then the assay mixture containing luciferin and luciferase was added and the luminescent was measured after 5 min incubation in RT.

### 4.6. Glycolytic Rate Measurements (Seahorse)

Glycolytic rate of MCF7 and MDA-MB-231 cancer cells was measured using Seahorse XF96 extracellular flux analyzer and a glycolysis rate kit (Seahorse Biosciences, Billerica, MA, USA). All the seahorse experiment has been performed in the absence of CO_2_/HCO^3−^. Oxygen consumption rate (OCR) and extracellular acidification rate (ECAR) of cancer cells were determined by seeding them on XF96 microplates in their growth medium until they reached over 90% confluence. In these studies, seeding started with 20,000 cells (80% of the well area). Measurements were determined 24 h later when the cells reached 90% confluence. One hour before the seahorse measurements culture media were removed and cells were washed 3 times with PBS followed by the addition of base medium (non-buffered DMEM supplemented with 25 mM glucose). Finally, data were normalized for total protein content of each well using the Bradford protein assay (ThermoFisher, Waltham, MA, USA). Seahorse measurements were performed with 4–6 technical replicates and these experiments were repeated at least 2 times. The WE phenotype (“Warburgness”) can be expressed as the ratio of glycolysis (ECAR) to oxidative phosphorylation (expressed as the oxygen consumption rate, OCR) from the GST.

### 4.7. Metabolic Profiling

Cells were seeded in a regular 96-well plate for 2D culture and in U bottom 96 well plates for 3D spheroid culture in their growth medium containing 10% FBS under standard culture condition. Once cells reach 90% confluence for 2D or caring capacity for 3D (usually when the growth of spheres is steady and they don’t get bigger), the growth media were removed and placed in a new 96 well plate with the same order as the original plate for metabolic profiling using YSI machine (YSI 2900 multi-analyte system (YSI, Yellow Springs, OH, USA)). We used at least 150 μL of media in each well to make sure there was enough media for each assay in the machine. Sensors in YSI were changed weekly and calibrated before each experiment. Final data of the lactate, glucose, glutamine, and glutamate in conditioned media were normalized to the protein amount per well or the confluency of each well imaged right before metabolite measurements. 

### 4.8. MCT Activity

Cells were plated on four chamber Lab-Tek chambered cover glass slides 1 day prior to experimenting, and cultured in RPMI media containing 10% FBS and 1% P/S. On day of experimentation, media was replaced with RPMI containing 10% FBS, 1% P/S, 25 mM HEPES, and 25 mM PIPES, to stabilize pH while outside of the 5% CO_2_ incubator. Once the assay was ready to begin for a given group, 5-(and-6)-Carboxy SNARF^TM^-1, Acetoxymethyl Ester, Acetate was added 8 min prior to experimentation at a concentration of 10 μg/mL. Prior to experimentation, the pH calibration curve of the 5-(and-6)-Carboxy SNARF^TM^-1, Acetoxymethyl Ester, Acetate dye was conducted using the nigericin method as described previously [36]. Once the 8 min incubation period was up, imaging began and superfusion with the 30 mM lactate solution (solutions listed in extended data file) and the SNARF-1 ratio was allowed to stabilize. The superfusion setup used in our experiments consists of a peristaltic to flow the media, a custom manufactured fluidic switch system which allows for the rapid changing of solutions, and a vacuum pump to aspirate media from the opposite side of the chamber. Once stabilized, cells were instantaneously switched to the 0 mM lactate solution and maintained in such until the velocity of the SNARF-1 ratio began to level off. Cells were then switched back to the 30 mM lactate condition and allowed to stabilize again. Once stabilized, the cells were switched to the same 30 mM lactate solution, this time either containing (X concentration of Diclofenac or Aspirin). Cells were maintained in the drug treatment solutions for 20 min in order for the drugs to take effect, and then switched to the 0 mM lactate solution containing the same concentration of drug they were treated with. 

### 4.9. pHi Measurement

Cells were plated on four chamber Lab-Tek chambered cover glass slides 1 day prior to experimentation and cultured in RPMI media containing 10% FBS and 1% P/S. On day of experimentation, media was replaced with RPMI containing 10% FBS, 1% P/S, 25 mM HEPES, and 25 mM PIPES, to stabilize pH while outside of the 5% CO_2_ incubator. Once the assay was ready to begin for a given group, 5-(and-6)-Carboxy SNARF^TM^-1, Acetoxymethyl Ester, Acetate was added 10 min prior to experimentation at a concentration of 10 μg/mL. After the 10 min, cells were washed 2 times with drug containing media and fresh drug containing media was resupplied to the cells. Change in pHi caused by each drug was measured by converting the SNARF-1 ratio to pHi and comparing pre- and post- treatment pHi’s. 

### 4.10. Spheroid Mono- and Co-Culture

Perfecta3^®^96-Well Hanging Drop Plates or non-adhesive U shape bottom 96 well plates were used to grow the primary spheres containing 10,000 total cells. 10% Matrigel was used for the primary spheroid construction as follows. After cells were counted they were diluted as 200 cells per μL of media. Then the cells mixture was cooled down in ice for 10 min and 10% Matrigel (melted in ice in the 4-degree Celsius walk-in fridge overnight) was added and directly seeded in the U plates. The plates were centrifuged for 1 min 500 RPM. For each experimental condition, the ratio between MCF-7-GFP and MDA-MB-231-RFP was 0, 50, or 100 percent as follows: 0/100, 50/50, 100/0. Each ratio had 4–8 replicates for each experiment. An Incucyte microscope kept at 37 °C and 5% CO_2_ was used to image the spheroid growth every 6 h over approximately 30 days. Images and fluorescent intensity were analyzed in Incucyte built-in software and image J. Relative fluorescent units (RFUs) were normalized by averaging red/green fluorescence when the corresponding cell type was absent then subtracting this value from other fluorescent values of that cell type. Drug treatments were directly added to the growth media of the spheroids and renewed every 3 days.

### 4.11. Animal Experiments

MCF7 and MDA-MB-231 breast cancer cell lines were grown in T-225 flask and harvested at 70–80% confluency. Both cells were authenticated by short tandem repeat analysis and tested for mycoplasma. 10 million cells in 200 μL cold PBS and 10% Matrigel was inoculated into cleared mammary fat pads of SCID mice (eight- to ten-week old females). Tumor measurements were done by calipers every other day and ultrasound once a week. Mice had free access to water and food for the whole duration of the experiment. For mixed cultures, a 1:1 mixture of 5 million MDA-MB-231 and 5 million MCF7 cells were injected. One week before cell injection, an estrogen pellet (0.72 mg slow-release, Innovative Research of America) was implanted to allow for the growth of ER-positive MCF7 tumors. The concentration of diclofenac given to each mouse was 40 mg/kg. As for the KA the concentration was 1 mg/kg. These concentrations remained the same when the mice were treated with both drugs. The control mice were injected with DMSO in PBS. The mice were treated by intraperitoneal injections once on Monday, Wednesday, and Friday weekly. At the end of the experiment tumors were extracted, then the size and weight of them were measured. Vital organs were also extracted and looked for metastasis.

Animal studies were performed by the guidelines of the IACUC of the H. Lee Moffitt Cancer Center (that was approved by the University of South Florida IACUC committee: IACUC 5331R).

## 5. Conclusions

We propose a novel strategy of cancer control for the tumor when a cure is not an option. We propose evolutionary principles of tumor growth can be used to control tumor cells’ clonal or sub-clonal population in favor of slower growth and less damage to patients. We showed that slight metabolic adaptations acquired during the evolution of cancer cells such as increased pHi can be used as vulnerability against them.

## Data Availability

All data presented in this study and not included within the supplementary materials are available by request submitted to the corresponding author.

## Supplementary Materials

The following are available online at https://www.mdpi.com/2072-6694/13/1/64/s1, Figure S1: Glutamine and glutamate concentration in conditioned media of MCF7 and MDA-MB-231 cells treated with diclofenac, Koningic acid, and both compared to non-treated control in hypoxia (0.1% oxygen) and normoxia, Figure S2: ICC Analysis of MCT1 and MCT4 expression in breast cancer cell lines. Figure S3: Extracellualr pH measurements of cells exposed to various treatment conditions. Treatment with KA, DIC, or combination of both results in an increase in extracellular pH. Figure S4: Survival assay of breast cancer cell lines in different microenvironmental conditions. Targeting MCTs and GAPDH under different microenvironments reduces the cancer cells’ viability. The effect is the most with the combination of both drug and under the most unique condition of solid tumors, hypoxia, and acidic pH. Data is normalized to non-treated for each cell line in each condition and presented as mean with SD as error bars. Each experiment has 4 replicates, Figure S5: Mono- and co-culture of fluorescently tagged breast cancer cell line. In different microenvironmental conditions and with any initial ration of MCF7: MDA-MB-231, the more aggressive cells, MDA-MB-231 always win. The spheroids are grown in normoxia but considering the size of spheres that reaches to 1–2 mm we know that oxygen concentration can vary from normoxia at the surface of spheres to hypoxia in the center.
