# Supplementary material for: Targeting of Evolutionarily Acquired Cancer Cell Phenotype by Exploiting pHi-Metabolic Vulnerabilities
Source: Cancers (Basel). 2020 Dec 28;13(1):64. doi: 10.3390/cancers13010064 (PMC7795337; doi:10.3390/cancers13010064)
Supplement: Supplementary file 1 [file cancers-13-00064-s001.pdf]

cancers

MDPI

Article

Targeting of Evolutionarily Acquired Cancer Cell Phenotype by Exploiting pHi-Metabolic Vulnerabilities

Bryce Ordway [1], Michal Tomaszewski [1], Samantha Byrne [1], Dominique Abrahams [1], Pawel Swietach [2], Robert J. Gillies [1] and Mehdi Damaghi [1,3,*]

[1] Department of Cancer Physiology, Moffitt Cancer Center and Research Institute, Tampa, FL 33612, USA; bryce.ordway@moffitt.org (B.O.); michal.tomaszewski@moffitt.org (M.T.); samantha.byrne@moffitt.org (S.B.); dominique.abrahams@moffitt.org (D.A.); robert.gillies@moffitt.org (R.J.G.)
[2] Department of Physiology, Anatomy & Genetics, University of Oxford, Oxford OX1 3PT, England; pawel.swietach@dpag.ox.ac.uk
[3] Department of Oncologic Sciences, Morsani College of Medicine, University of South Florida, Tampa, FL 33612, USA
* Correspondence: Mehdi.damaghi@moffitt.org

Supplementary

Citation: Ordway, B.; Tomaszewski, M.; Byrne, S.; Abrahams, D.; Swietach, P.; Gillies, R.J.; Damaghi, M. Targeting of Evolutionarily Acquired Cancer Cell Phenotype by Exploiting pHi-Metabolic Vulnerabilities. *Cancers* **2021**, *13*, 64. https://doi.org/10.3390/cancers13010064

Received: 14 November 2020
Accepted: 23 December 2020
Published: 28 December 2020

Publisher's Note: MDPI stays neutral with regard to jurisdictional claims in published maps and institutional affiliations.

Copyright: © 2020 by the authors. Licensee MDPI, Basel, Switzerland. This article is an open access article distributed under the terms and conditions of the Creative Commons Attribution (CC BY) license (http://creativecommons.org/licenses/by/4.0/).

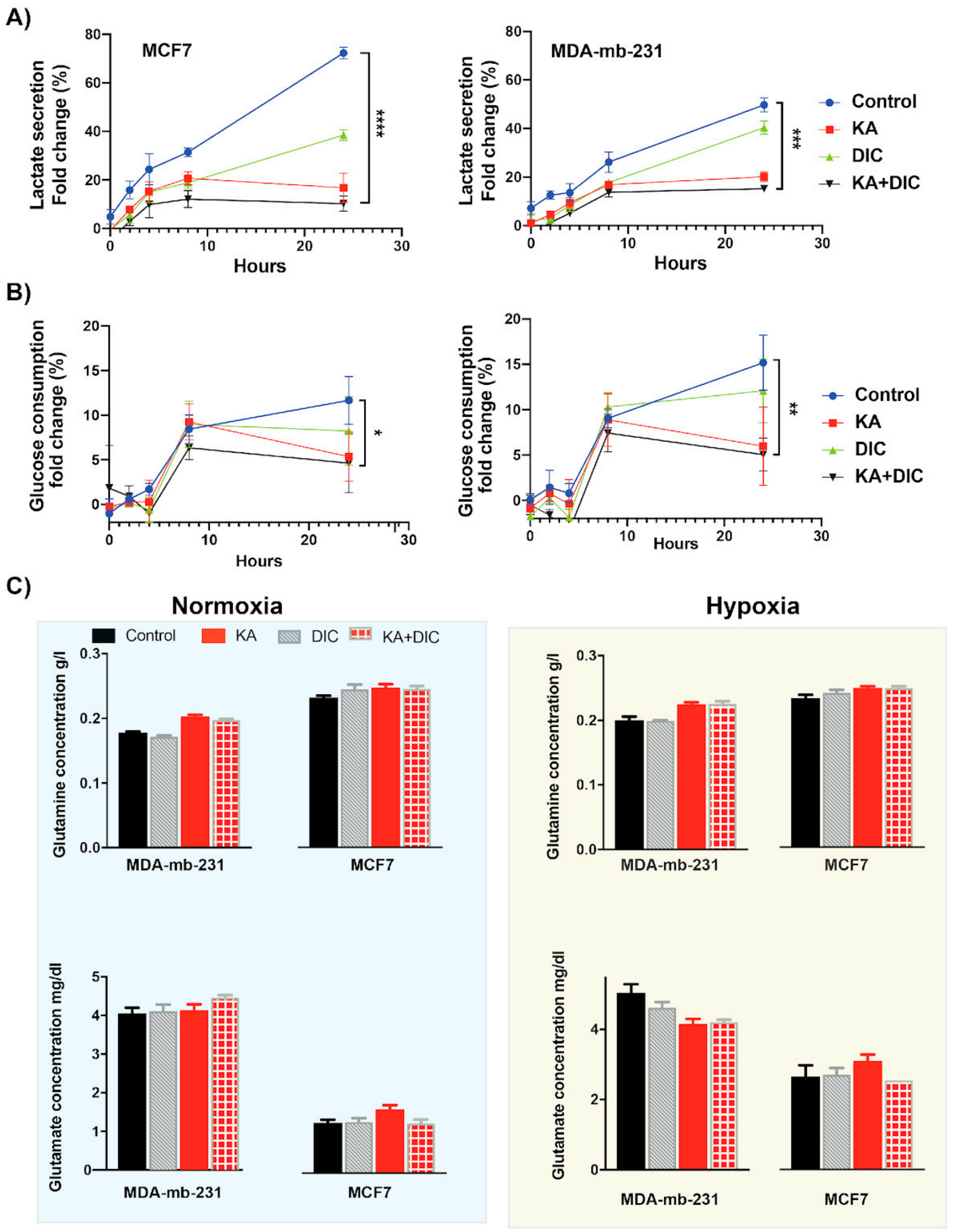

Figure S1. Glucose consumption was reduced in both MCF7 and MDA-mb-231 cells after 72 hours treatment with diclofenac, KA, or both. (**A**) Time point measurement of lactate production in both MCF7 and MDA-mb-231. (**B**) Time point measurement of glucose consumption in breast cancer cell lines. Data are represented as mean with SD as error bars. (**C**) Glutamine and glutamate concentrations in conditioned media of MCF7 and MDA-mb-231 cells treated with Diclofenac, Koningic acid, and both compared to the non-treated control in hypoxia (0.1% oxygen) and normoxia. p-values are represented as follows: * $p < 0.05$, ** $p < 0.01$, *** $p < 0.001$, **** $p < 0.0001$.

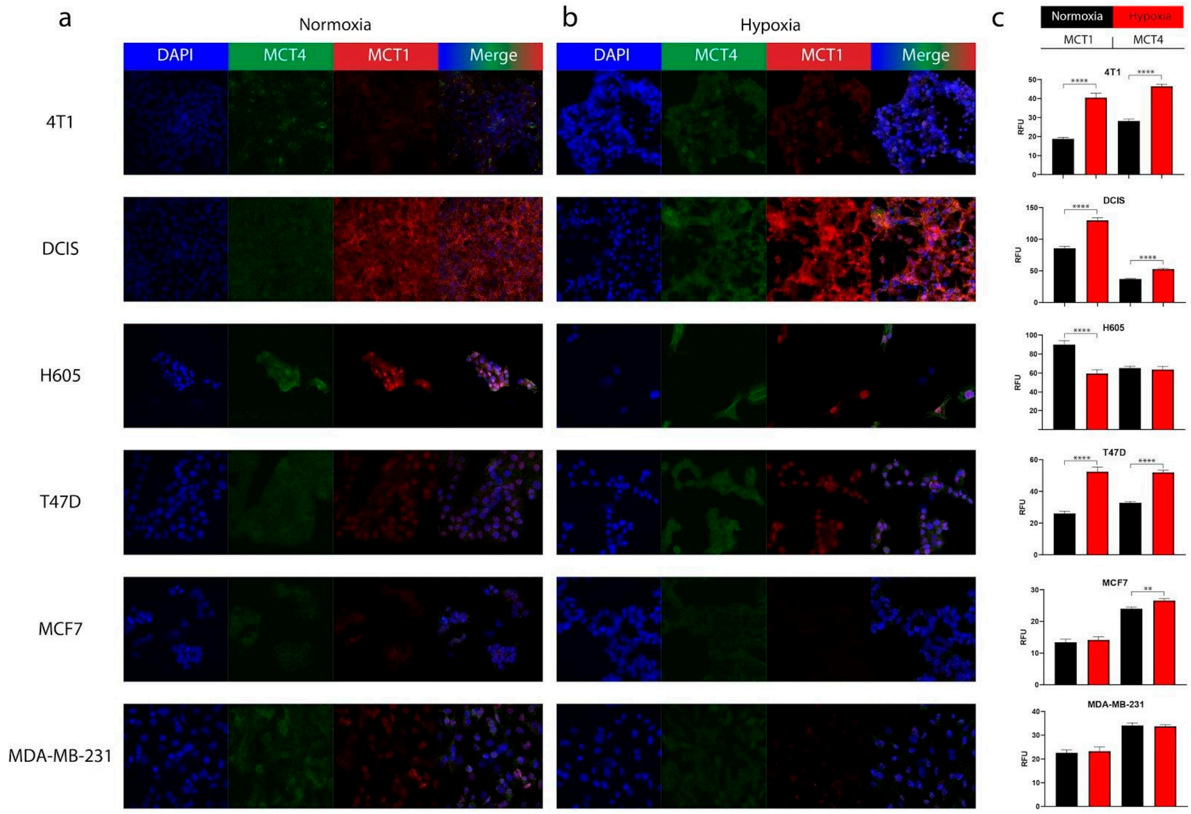

Figure S2. ICC Analysis of MCT Expression in Breast Cancer Cell Lines. ICC staining and analysis of 4T1, DCIS, H605, T47D, MCF7, and MDA-MB-231 cell lines. (**a**) DAPI, MCT4, and MCT1 staining of aforementioned breast cancer cell lines under normoxic oxygen conditions. (**b**) DAPI, MCT4, and MCT1 staining of aforementioned breast cancer cell lines under hypoxic oxygen conditions. (**c**) Quantitative analysis of ICC staining. *p*-values are represented as follows: * $p < 0.05$, ** $p < 0.01$, *** $p < 0.001$, **** $p < 0.0001$.

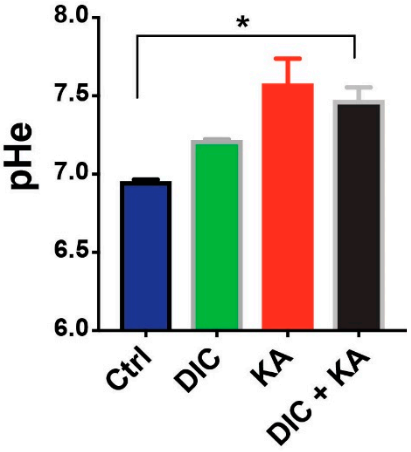

Figure S3. Extracellular pH measurements. Media pH of cells treated with diclofenac, KA, or combination of both. *p*-values are represented as follows: * $p < 0.05$, ** $p < 0.01$, *** $p < 0.001$, **** $p < 0.0001$.

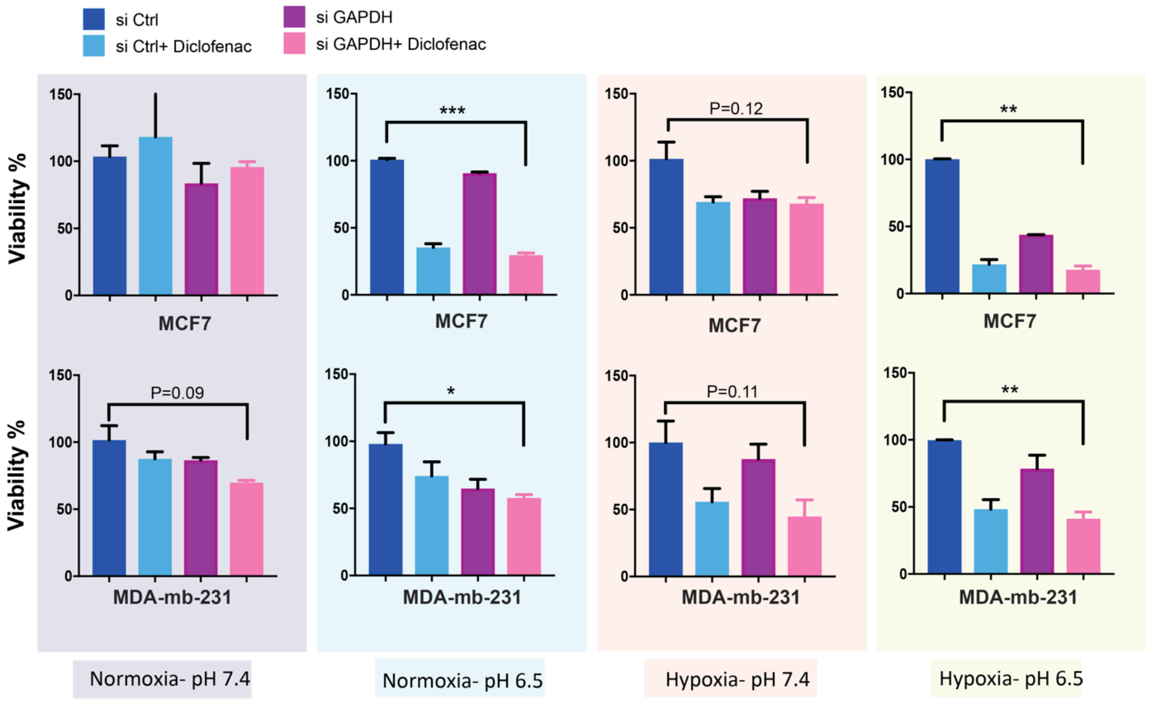

Figure S4. Survival assay of breast cancer cell lines in different microenvironmental conditions. Targeting MCTs and GAPDH under different microenvironments reduces the cancer cells' viability. The effect is the most with the combination of both drugs and under the most unique condition of solid tumors, hypoxia, and acidic pH. The data is normalized to non-treated for each cell line in each condition and presented as mean with error bars as SD. *p*-values are represented as follows: * $p < 0.05$, ** $p < 0.01$, *** $p < 0.001$, **** $p < 0.0001$.

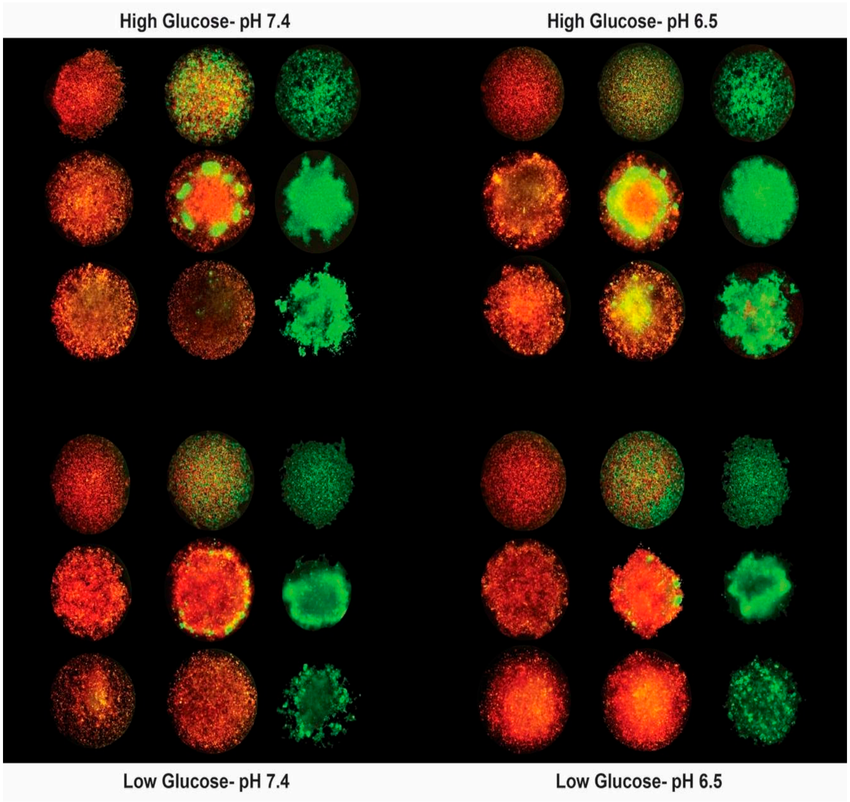

Figure S5. Mono- and co-culture of fluorescently tagged breast cancer cell lines. In different microenvironmental conditions and with any initial ratio of MCF7-GFP: MDA-MB-231-RFP, the more aggressive cells, MDA-MB-231 always win. The spheroids are grown in normoxia but will have hypoxia at the center; considering the size of spheres that reach 1–2 mm, we know that the oxygen concentration can vary from normoxia at the surface of spheres to hypoxia in the center.
